# Patterns as basis for immersivity across the arts: a practice-led hypothesis

**DOI:** 10.3389/fpsyg.2025.1670384

**Published:** 2025-08-29

**Authors:** Hedvig Jalhed

**Affiliations:** ^1^Academy of Music and Opera, School of Education, Culture and Communication, Mälardalen University, Västerås, Sweden; ^2^Malmö Academy of Music, Faculty of Fine and Performing Arts, Lund University, Malmö, Sweden

**Keywords:** immersivity, immersion, emersion, arts, games, cognition, pattern processing

## Abstract

This article introduces the pattern theory of immersivity (PTI), a practice-led theoretical framework stemming from the hypothesis that a reorientation of the understanding of immersive art is needed, away from technological sophistication or sensory excess and toward the psychological mechanisms underlying imaginative engagement, regardless of artform or combination of media. The theory posits that immersivity—the capacity of an artwork to induce immersion—is primarily a function of patterned information structures that stimulate aspects of superior pattern processing (SPP). Immersion is reconceptualised as a process of cognitive filling-in, in which motifs that remain partially concealed elicit fantasies. A distinction is made between immersive perception and immersive performance, each articulated across three graduated levels, culminating in a peak immersive state. This pan-artistic model, formalised as the ORFEUS model, offers artists a tentative tool for designing and analysing the prerequisites for immersive experiences through strategic use of fragmentation, ambiguity, and indirect information. The theory underscores that immersivity is seldom a property of entire artworks, but rather a quality embedded in specific elements that provoke psychological engagement. Ultimately, PTI provides a refined vocabulary and a methodological approach for immersive artmaking in all media; one that integrates artistic know-how with knowledge about the deep structures of human cognition.

## 1 Introduction

Artistic practitioners aiming to induce immersion must understand the underlying psychological and cognitive processes at play and distinguish these from other mental states that may produce similar or interrelated responses. However, from the artist’s perspective, I highlight that it is *immersivity*—here understood as the capacity of an information arrangement to foster immersion—rather than the mental state or behavioural condition of immersion per se, which warrants closer examination. This shift in focus calls for an approach to artmaking as a form of applied psychology. We must ask: *What drives and maintains immersion, thus forming the basis of immersivity?*

Grounded in multisensory art, my practice-led hypothesis proposes that we should attend not primarily to technical performance or sensory impact of supposedly immersive art, but to the motifs embedded in the artworks and the motivations of the perceivers and performers. I suggest that what we become immersed in is not simply ‘spaces’, ‘worlds’, or fantasies, but *patterns*. These patterns can and do indeed spark imagination. But what differentiates immersive experiences from merely imaginative ones, I suggest, is their connection to supporting information structures that activates certain cognitive biases while simultaneously dampening self-consciousness. The purpose of this article is to provide artists with a revised theoretical framework, what I call the *pattern theory of immersivity* (PTI). PTI defines immersivity as the capacity of an information structure—not a medium or technology—to foster immersion. Central to this reconception, applicable across the arts, is the idea that the human brain has evolved to seek, detect, and complete patterns through what Mattson (2014) highlights as our capability for *superior pattern processing* (SPP), emerging with our expanded cerebral cortex. This capacity is linked to high-level cognitive abilities such as language, imagination, and even belief in supernatural entities. Immersive art, in this view, is effective when it activates SPP through partial information, inviting audiences to imagine what is obscured. And this neurobiological approach does not negate the role of sensory input in immersive works—it reframes it.

Immersion and immersivity are topics with relevance for multiple scientific and artistic domains, from media studies, cognitive science, and psychology to worldbuilding, interaction design, staging, and storytelling. In art and entertainment, immersion is a widely used but inconsistently defined term across disciplines like theatre, music, gaming, and literature. Efforts to explain immersion as a mental state and a reported feeling triggered by artistic or ludic input has generated different conclusions, emphasising media’s and technology’s abilities either to stimulate the immersees’ cognitive absorption or to overflow their sensory faculties (Carr et al., 2006; Jennett et al., 2008; Jennett et al., 2009; Wolf, 2017; Paananen et al., 2024). Although sought across all arts, and in the artistic realm commonly understood to be in line with Grau’s (2003, 13) definition as something “characterised by diminishing critical distance to what is shown and increasing emotional involvement in what is happening”, conceptualisations of immersion have come to vary between different arts and media. In immersive theatre, the beholder’s participative, non-distanced position and openness to become involved have become core features (Machon, 2013; Alston, 2016). In immersive music, immersion has been presumed to be a function of 3D audio, embedding the listener in atmospheric, ambient, and spatialised sounds (Wycisk et al., 2022). In immersive narratives, evasion of touching upon the relation between the actual and the fictional and avoidance of attention-shifts between meta-level framework and imaginary content are regarded as immersive (Ryan, 2001; Mendlesohn, 2008).

The different conceptualisations of what is supposed to constitute essentials in immersive art practices share a set of ‘family resemblances’, but their breadth risks leading to inconsistent frameworks that dilute the concept rather than ground it in shared principles. Such principles are especially important in interdisciplinary artmaking, which combines different practices and media. For some, such as actors and writers, immersion centres the enchantment of the beholder, while for others, like musicians, the envelopment of the audience is the core focus. But although opportunities for adventurous roaming and close contact with actors can be exciting, and being surrounded by powerful loudspeakers can be overwhelming, not all such experiences are immersive in the same way as some deeply stimulating novels, movies, and computer games. However, some are, and my hypothesis—that *what* the artwork implies is more crucial for its immersive impact than *how* it is technically set up—does not dismiss the influence of either interaction in fiction or sensory input. Rather, it approaches the issue from a new angle, urging us to consider the potential role of thematic content in immersive art in relation to basic functions of the human psyche—specifically, SPP.

The shift in perspective can be highlighted by how we use the metaphor ‘immersive’, which etymologically is derived from the Latin *in mergere* = to *merge in* or to *sink in*—to *submerge*. Immersion is generally described as occurring when the human mind is filled outside-in, like an absorbing sponge drenched in liquid. The alternative view that I suggest conceptualises immersion as the act of imaginatively filling in the gaps in muddled and labyrinthic patterns of missing information, no matter whether the information is transmitted visually, audially, or verbally. To be immersed is, in this view, to pour our own imagination into the given framework. That makes immersivity into a matter of *information porosity* and immersive arts and games into stimulating challenges for the imagination rather than pure and indulgent services or side effects. This approach bears some similarities to how Agrawal et al. (2020) proposes a distinction between *immersive potential* (the potential of a system or content to elicit immersion) and *immersive tendency* (an individual’s predisposition to experience immersion). It also follows that an immersive pattern can generate slightly different subjective results depending on the fantasist’s mood, age, education, cultural references, and so on, but also that there probably are general templates for pattern detection from evolved psychological mechanisms connected to SPP. What remains non-immersive are flattened, open, and straight-forward patterns of information.

What I will argue is that cognitive absorption—in this reversed version in which it is the artwork that is a ‘sponge’ waiting to be soaked and vivified with our hopes and fears—and sensory overflow work in supplementary ways in immersive art. Immersivity is also a matter of hindering or detaining *emersion*, which is the counterpart to immersion (Kubinski, 2014). I will further stress the importance of distinguishing the cognitive processes of immersion from those of *flow*, as conceptualised by Csikszentmihalyi (1990), even though flow can be nested within immersive contexts. While immersive practices encompass both sensory and cognitive dimensions, I contend that their potency resides in how they provide us with patterns for the imagination to comprehend and complete, so that what Machon (2013) characterises as visceral, embodied responses are evoked. Rather than centring on the sensuous impact of our practical achievements and embodied responses when interacting with tools and toys, PTI suggests that immersive art shifts our awareness to the figments of imagination we project onto the patterns that inspire them.

## 2 Narrowing the scope

From the artist’s perspective, it is important to draw several distinctions to better define the problem of immersivity and navigate the fuzzy boundaries between immersivity and related concepts that evoke similar or overlapping states of fascination and involvement. I am aware of the challenges of disentangling these interwoven experiences. Nevertheless, it is precisely these nuances that matter most for my line of reasoning.

First, immersivity is not simply the capacity to gain and hold the attention of viewers, listeners, or readers. Many artworks are impressive, compelling, or impactful—and therefore engrossing, enjoyable, or engaging—without necessarily being immersive. The professed immersive artist, therefore, cannot solve the problem merely by addressing what drives and sustains attention.

Second, if immersion occurs through the exercise of imagination within a given framework, we must not confuse imagining with interpreting. Comparing different readings of immersive works or applying hermeneutics tends to inhibit the immersive mindset, since immersivity, if connected to imagination, aims to activate subjective experience, not objective analysis, meta-perspectives, or self-reflexivity (Civitarese, 2017). The end-product of immersive art is thus the intensity and memorability of the fantasy element sparked by the immersive structure—something that can only be attained and retained through introspection. For a more detailed discussion of the distinctions between fantasising and imagining in relation to art, see Stock (2009).

Third, although we often speak of being immersed in a fiction or fantasy, it is entirely possible to imagine without being immersed; there is not necessary any causal link between the two. Immersion can be discussed in relation to belief (Schellenberg, 2013), and some might argue that immersion depends on the depth of one’s involvement with the fantasy—dreaming, in that sense, being the ultimate immersion. However, if we instead consider immersive fantasy to be a function of external input that provides us with information including immersive qualities or potential, it is not a matter of being open to fantasy and ideas but being able to make inferences based on certain arrangements. The question then becomes which features are capable of triggering the fantasies we find in immersive experience.

Fourth, even though it can certainly be combined with immersive structures, interactivity is not, per se, an immersive quality. While many games and game-based artworks are described as immersive, their appeal often lies primarily in their interactivity and the positive experience of flow they can induce through tasks offering optimal challenges. A difference between immersion and flow states is that immersion also can include negative emotions and anxiety (Jennett et al., 2008). Furthermore, statistical or opinionated forms of interaction—such as audience polls, commentaries, or other direct feedback—do not, in themselves, activate imagination in relation to the information provided, and thus should not be considered part of immersive practice when discussed in this particular psychological sense.

The starting points for my continuing argument are established by demarcating immersion from impression in this way, and underscoring the distinct functions of imagination, interpretation, and interaction in relation to immersive art.

## 3 Sensory stimulation of cognitive biases

If immersion should not be conflated with sensory impression in general, we must examine the specific functions sensory stimulation serves in immersive art under this narrower definition. If a core function is to stimulate imagination rather than interpretation, a theory of immersivity should at least tentatively account for how this can be achieved in practice. To move beyond media-specific conceptualisations of immersive features and toward a pan-artistic theory of psychological stimulation, the focus shifts from aesthetics and design to *pattern theory*. Advocating for applied pattern theory and pattern thinking more broadly—while also raising questions about how artists engage with patterns—Ellaway (2025, 76) observes that “[a] common quality of artistic and athletic performances that make them enjoyable is a sense of uncertainty within the patterns they embody; a performance that has unexpected consequences, a plot twist or new take on an old idea, anything that both complies with a pattern and yet still innovates and confounds expectations.” In immersive art, however, the unexpected is not enough—we must also produce instantiations of the seemingly unknown to evoke fantasy, not merely suspense. And the arts are capable of activating our evolved capabilities of SPP and present fabricated patterns for imagination and magical thinking, but also circumjacent structures that might challenge the distinction between reality and fantasy (Mattson, 2014).

In relation to the surrounding environment, sensory input can either draw attention to select information that sparks fantasy or shield us from disruptive stimuli. The strange, wonderland-like mood of immersion is fragile, easily broken by reminders of ordinary life and ‘wake-up calls’ pulling us back to our regular mode of being. In immersive settings, we are momentary guests in strangeness, and the job of artists and authors is to withhold information and add confusing elements and embellishments. If we speculate that immersion in strange or stylised information requires suppressing our habitual mode of perception, we might find an analogy in language immersion: bilinguals immersed in a less-dominant language momentarily inhibit the dominant one (Zhang et al., 2021). Similarly, immersion in fiction is intertwined with a lack of familiarity (Anderson and Iversen, 2018), and what distinguishes artful immersion is often the feeling of fragmented understanding—a fluid mix of moments of clarity and stretches of obscurity—unlike the coherence of more familiar and predictable contexts. Sensory input sustains immersion either by blocking external distractions, as in enclosed haunted houses, escape rooms, or 360-degree domes, or by keeping us engaged in imaginative activity. When immersed in this sense, our attention turns inward, toward our own responses to the fantasies ignited by the structures surrounding us.

Fantasies involving unfamiliar realms and largely unknown or mysterious beings—including fantasy creatures or alien life—are a recurrent theme in immersive experiences. And the most basic way immersive art uses patterns to evoke fantasy may lie in how it makes us respond to the patterns of life itself. Humans are especially prone to infer animacy and agency from perceived patterns—a cognitive bias that immersive art often exploits.

The detection of patterns of animacy is central to both human and non-human animals, playing a vital role in directing attention to potential predators or partners. Recognition of animacy is not based solely on appearance, but also on dynamic motion cues. Research shows that human infants can distinguish between animate and inanimate motion from an early age, and display a marked preference for animate motion patterns (Lunghi and Di Giorgio, 2024). This attentional bias likely has deep evolutionary roots (Öhman et al., 2001; New et al., 2007). Artistic representations that incorporate animate or quasi-animate motifs—or patterns that imply animate motion—tap into this innate predisposition.

Agency detection in humans involves the attribution of beliefs, emotions, attitudes, knowledge, and intentions to other agents. Because agents act with goals and purposes, we predict their behaviour by projecting mental states onto them—memories of the past, aspirations for the future, and so on. This capacity is adaptive, enabling us to combine attention paid to external actions with assumptions about internal states. Yet our hypersensitivity to patterns of agency often leads us to over-attribute its meaning, perceiving intentionality where none exists (Kühnen and Kitayama, 2020). As the attribution of mental states includes mentalisation or ‘theory of mind’ (ToM), patterns of intentional and emotionally driven actions can be used conversely by actors and directors who wish to hint at a certain character of thought or motivation. Likewise, *apophenia*, or the human tendency to perceive connections and meaningful patterns among unrelated or random data, can be utilised in art. Associated with hypnagogic and psychotic states [see Conrad in Mishara (2009)], apophenia may be understood as an exaggerated form of a natural cognitive inclination. Shermer (2008) has coined the term *patternicity* to describe this phenomenon in a broader context, encompassing everything from paranormal beliefs to conspiracy theories. In art, the power of patternicity lies in its generative ambiguity. I suggest that this inclination toward apophenia and patternicity also helps explain the dreamlike, quasi-somnambulic state sometimes observed in deeply immersed individuals who are connecting the dots in fabricated structures.

Through hints and clues, immersive artists can fuel fantasies of prey and predators, friends and foes, beauties and beasts. These are not shown directly but suggested through attributes. Immersive art, I argue, works through indirect and incomplete information. This is what distinguishes a movie like *Jaws* from a nature documentary about sharks or the computer game *Myst* from a recreational or therapeutic VR experience of an island vacation. It is not the medium that makes an artwork immersive, but how thematic and patterned information is used as a psychological feature.

Following this line of reasoning, physical stimuli in immersive art should primarily suggest the signals and effects of creatures moving through the world rather than reveal the creatures themselves—at least up to a point. It is furthermore probably not a coincidence that many immersive events take place at night-time or during twilight, and not in broad daylight when disturbances and distractions are more pronounced. Also, institutional immersivity produced through the still, silent, and dark auditorium, concert hall, or library has a mystifying and fantasy stimulating character, as do campfires beneath the stars. From the performing artist’s perspective, the live but ghostly restrained audience in the shadowy hall is extremely immersive; artists can also be immersed themselves. Furthermore, misty landscapes, shadowy silhouettes, obscured features, approaching movements, discarded belongings, and traces of past actions prompt the mind to infer more than it perceives directly. The reactions of characters, as well as their stories about what they perceive or have experienced, serve the same purpose. As Lukka (2014) has observed as an immersive factor in the case of role-playing, we are even able to imagine ourselves as another being by shifting our perspective to see the world through that being’s viewpoint—perhaps, the viewpoint comes first and the character second. This mechanism operates in literature, film, and games, allowing us to follow the patterns of reaction that arise from such a manoeuvre. Even though disassociation from the outside world is commonly viewed as a constituent of immersion, it is perhaps rather disconnection from ourselves which merits discussion, as many immersive events allow us to just navigate ordinary life as if we were someone else.

## 4 Immersive perception and performance

I recapitulate the core claims of the pattern theory of immersivity as developed thus far: immersive constructs stimulate fantasy through patterns of incomplete information. These patterns often relate to fantasies of living beings—whether mythological, feral, or alien. Crucially, such beings remain immersive only insofar as they are not fully known; information about them is indirect and they are rendered perceptible primarily through patterns of past and current motion—their movement patterns—which lends them an eerie, elusive quality. I now turn to the distinction between *immersive perception* and *immersive performance*, further elaborating on their interrelation, as articulated by Jalhed (2022).

Perceivers in immersive situations occasionally find themselves invisible ‘flies on the wall’, able to see, hear, and understand in ways that they normally could not, or encounter signs of irregular behaviour. I outline three gradations of immersive perception, each situated within a context where the perceiver is either a visitor in an unfamiliar environment or confronted with an unfamiliar element entering their own space (cf. Mendlesohn, 2008):

1. **Organisation**–the discovery of a habitat and the signs of patterns of organised movement
2. **Routinisation**–the recognition of habits and the discernment of routine movement patterns
3. **Focalisation**–the perception of how a habitant’s focalised movements patterns provoke action and how their subjective point of view relates to information sources and objects

From these levels, anticipatory fantasies naturally emerge: What has been prepared or planned? What customs or needs can be inferred? Who or what might be expected to show up? The central premise remains; immersive art communicate life not through exposition, but indirectly—through movement patterns. However, the anticipation derived from the capacities of SPP is of course not a simplistic process, since pattern perception is based on interlinked associations:

More prosaically, contextual pattern recall can help us with filling in the ambiguities and unknowns in perception; it is what constitutes the sense of familiarity in places, people, and activities we have encountered before. A series of interlinked cues can, therefore, precipitate a state of anticipation if the cues correspond and cumulatively suggest patterns that contain deeper understandings of a phenomenon. Anticipation is not simply associative; it is predictive and preparative. Predictive in the sense that it suggests that something is likely to happen (suggesting degrees of certainty) and preparative in the sense that relevant patterns needed to respond can be activated in advance of their being needed (Ellaway, 2025, 51).

While immersive perception forms the foundation of immersive impact and often stands on its own, I posit that immersive performance constitutes an additional and equally significant dimension. This notion of performance can, to some extent, be compared with the immersive aspects of incorporation, as suggested by Calleja (2011). However, whereas immersive perception involves the indirect discernment of movement patterns, immersive performance concerns integration into environment patterns that are more or less directly replicable, forming the basis for participatory fantasies. In immersive contexts, performers engage obliquely—rather than as their authentic selves—a dynamic central to the frequently observed phenomenon of dissociation in immersive experiences (Aardema et al., 2010; Jennett, 2010; Williams, 2014). At one end of the spectrum lies a diffused state in which the subjective perceiver/performer and the objective pattern remain distinct and separated; at the other, a camouflaged convergence in which the two seamlessly fit together. Corresponding to the three levels of immersive perception, I propose three gradations of immersive performance:

1. **Symbolisation**–the use of avatars, fictive characters, dolls, game pieces and other movable items as proxies, allowing participants to act through virtual or material marionettes
2. **Unitisation**–the assumption of a representative function or type, marked by affiliation and allegiance, acting on behalf of someone or as part of something
3. **Equalisation**–the most immersive form, in which participants blend into the setting in an embodied way, as integrated denizen personalities, spying or larping as if another individual

None of these performance modes encourage independent or idiosyncratic movement; instead, they scale in terms of sensory proximity in relation to the environment. This progression moves from the distanced and abstracted state of symbolisation, through the tuned embodiment of unitisation, to the near-total integration of equalisation, in which the performer blends into the immersive pattern. I propose that this convergence—where matter and pattern are totally adaptive and become confused in the highest degrees of both immersive perception (focalisation) and performance (equalisation)—constitutes the condition of peak immersion.

**Figure 1:**
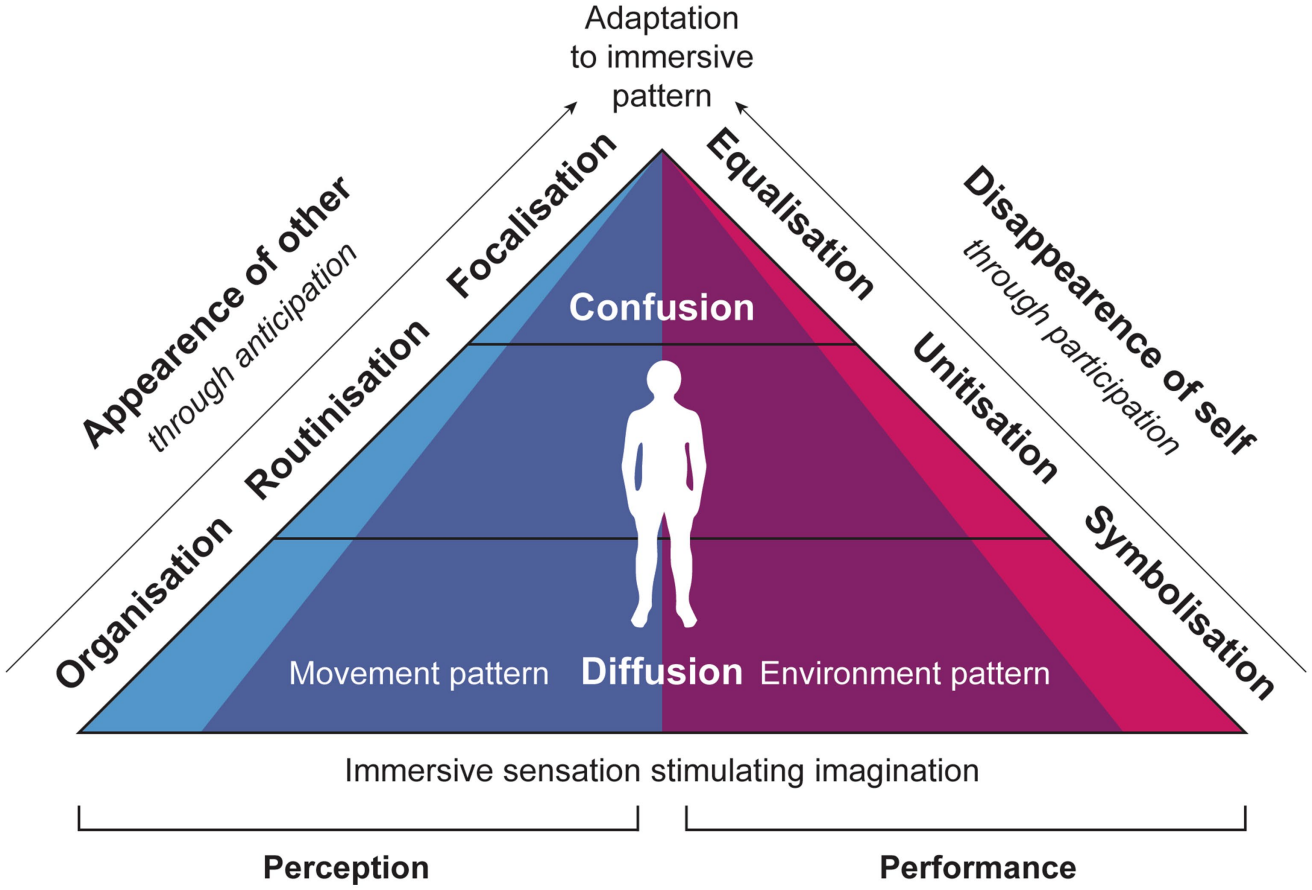
Diagram of the ORFEUS model with parallel gradations of immersive perception and immersive performance in relation to sensory stimulation of imagination, ranging from diffusion at the base to confusion at the top.

The model I propose for artistic application and analysis maps the levels of immersive perception and performance, and is titled the ORFEUS model, an acronym derived from Organisation, Routinisation, Focalisation, Equalisation, Unitisation, and Symbolisation (see Figure 1). It presents immersive perception as a process of *calculation* of the probable and immersive performance as a process of *calibration* of the proximate.

## 5 Basis for artistic application

One implication of PTI is the need to develop artistic strategies that affirm and intensify the gradual unfolding of patterns with affective resonance. What distinguishes immersive art from non-immersive forms is not merely its capacity to engage the senses, but the deliberate use of noise, fragmentation, and indeterminacy. Techniques that blur text, distort sound, or obscure key imagery become central tools in the immersive repertoire. To revisit the metaphor of submersion in liquid: the dim, filtered light and the muffled, inarticulate sounds experienced beneath the surface of murky water are instructive.

Sensory stimulation enhances immersive perception by supporting the levels outlined earlier regarding emergent movement patterns. It likewise supports immersive performance along a parallel continuum toward concrete environment patterns. In both cases, sensory input helps guide the participant’s attention away from ordinary business and toward a mode of speculative engagement.

According to PTI, efficient immersivity is achieved when patterned information gives rise to imagined expectations—when the unknown appears imaginatively animated—and when the perceiver/performer, in turn, becomes absorbed and, in a very tangible sense, ‘disappears’ into patterns. In short, *immersivity is when nobody is really there*; neither the perceived nor the performing.

## 6 Discussion

PTI advances beyond conventional understandings of immersive impact. By emphasising the role of recurring motifs in immersive art in relation to SPP—rather than focusing solely on technological domination of the senses—this theory reframes immersion as a product of patterns and immersivity as the capacity of displaying potent patterns. In doing so, it offers an alternative model in which immersive perception and performance unfold along a graduated continuum. The idea of patternmaking as an artistic activity is not new, but the idea of patterns as the driver of immersive experience has not been forwarded in this way.

One might argue that narrowing the definition of immersivity risks imposing a normative framework that could constrain artistic freedom by imposing rigid criteria. However, I argue that focusing on parts of works, rather than on whole experiences, allows for more nuanced critique and artistic reflection. This distinction renders a more precise and critical vocabulary: not all aspects of a film, a video game, or a novel are immersive, but certain components or elements—characters, motifs, sequences—may possess immersive qualities. For example, the elusive killer in *Twin Peaks*, the sought-for allies in *Baldur’s Gate*, and the mysterious woman in the attic in *Jane Eyre* all function as immersive creatures. By identifying such elements, we gain not only sharper theoretical tools but also a more rigorous basis for critique.

The indirectness and strangeness that define immersive perception and immersive performance lends immersivity a quality akin to artificial dreaming. In immersive spaces, participants are not present as their material selves but instead relinquish their regular behavioural patterns to transgress borders in a facilitated way and influence the space set up with artistically constructed patterns. Conversely, emersion is the act of breaking from these patterns—of interrupting promoted expectations and reasserting one’s own agency or potency. This interplay between immersion and emersion marks a dynamic tension at the heart of immersive art: the dreamlike fascination with cryptic patterns and the return to the directness and responsibilities of everyday life.

If PTI is found relevant to immersive art practice after more experimental research, basic knowledge about SPP and related mechanisms of human brain functions connected to how patterns are produced cognitively and creatively should be part of artistic education.

## Data Availability

The original contributions presented in the study are included in the article/supplementary material, further inquiries can be directed to the corresponding author.
